# Resistance Trends and Epidemiology of *Citrobacter*-*Enterobacter*-*Serratia* in Urinary Tract Infections of Inpatients and Outpatients (RECESUTI): A 10-Year Survey

**DOI:** 10.3390/medicina55060285

**Published:** 2019-06-18

**Authors:** Márió Gajdács, Edit Urbán

**Affiliations:** 1 Department of Pharmacodynamics and Biopharmacy, Faculty of Pharmacy, University of Szeged, Eötvös utca 6., 6720 Szeged, Hungary; 2 Institute of Clinical Microbiology, Faculty of Medicine, University of Szeged, Semmelweis utca 6., 6725 Szeged, Hungary; tidenabru@freemail.hu

**Keywords:** urinary tract infection, UTI, antibiotic, resistance, indicator, epidemiology, fosfomycin, ESBL, *Citrobacter*, *Enterobacter*, *Serratia*

## Abstract

*Background and objectives:* Urinary tract infections (UTIs) are the third most common infections in humans, representing a significant factor of morbidity, both among outpatients and inpatients. The pathogenic role of *Citrobacter*, *Enterobacter*, and *Serratia* species (CES bacteria) has been described in UTIs. CES bacteria present a therapeutic challenge due to the various intrinsic and acquired resistance mechanisms they possess. *Materials and Methods:* The aim of this study was to assess and compare the resistance trends and epidemiology of CES pathogens in UTIs (RECESUTI) in inpatients and outpatients during a 10-year study period. To evaluate the resistance trends of isolated strains, several antibiotics were chosen as indicator drugs based on local utilization data. 578 CES isolates were obtained from inpatients and 554 from outpatients, representing 2.57 ± 0.41% of all positive urine samples for outpatients and 3.02 ± 0.40% for inpatients. *E. cloacae* was the most prevalent species. *Results:* The ratio of resistant strains to most of the indicator drugs was higher in the inpatient group and lower in the second half of the study period. ESBL-producing isolates were detected in 0–9.75% from outpatient and 0–29.09% from inpatient samples. *Conclusions:* Resistance developments of CES bacteria, coupled with their intrinsic non-susceptibility to several antibiotics, severely limits the number of therapeutic alternatives, especially for outpatients.

## 1. Introduction

Urinary tract infections (UTIs) are the third most prevalent type of infections in human medicine worldwide, following respiratory and gastrointestinal infections, while in Europe, UTIs are the second most prevalent type of infections in humans [1,2]. UTIs are a significant factor of morbidity, both among outpatients and hospitalized patients [3]. In fact, hospital acquired UTIs are the most common healthcare associated infections (i.e., nosocomial infections). They account for 25–50% of nosocomial infections overall, representing a serious economic and public health issue for healthcare institutions [1,2,4]. UTIs are most commonly caused by members of the *Enterobacterales* order (typical pathogens include *Escherichia coli* and *Klebsiella* spp.), however, several bacteria, which were previously isolated infrequently (e.g., the *Proteus-Providencia-Morganella* tribe, *Citrobacter-Enterobacter-Serratia* species) have now emerged as increasingly relevant pathogens in UTIs, both in community and nosocomial settings [1,2,3,4,5,6].

Species of the *Citrobacter*, *Enterobacter*, and *Serratia* genera (hereafter abbreviated as *CES*) are facultative anaerobic, non-spore forming Gram-negative bacilli. They are widely distributed in the environment (soil, water) and in the gastrointestinal tract of animals and humans [7]. Discussion of these three genera together is justified by their similar biochemical characteristics, prevalence, and resistance trends [8]. The pathogenic role of CES bacteria has been described in urinary tract infections, respiratory tract infections, bacteremia and sepsis, gastroenteritis, conjunctivitis, wound infections, endocarditis, meningitis (both in adults and neonates), and brain abscesses [2,9,10,11,12,13,14,15,16,17,18,19,20,21]. In recent years, outbreaks associated with CES bacteria has become more frequent (especially in neonatal and adult intensive care units), highlighting that these bacteria pose a serious concern from an infection control perspective [9,10,11,12,13,14,15,16,17,18,19,20,21].

Compared to *E. coli*, members of CES are more frequently isolated in complicated UTIs (associated with catheters, functional or anatomical abnormalities of the genitourinary tract) from patients with underlying conditions or immunosuppression. They are also more frequently associated with pyelonephritis, recurrence, and prolonged therapy [9,10,12,13,14,15,18,20,21]. CES bacteria present a challenge to clinicians and microbiologists alike due to the various intrinsic and acquired resistance mechanisms they possess. They are all intrinsically resistant to penicillins, several β-lactam/β-lactamase combinations (e.g., ampicillin/sulbactam, amoxicillin/clavulanic acid), first–second generation cephalosporins, and cephamycins (i.e., cefoxitin), due to their penicillinases and AmpC-β-lactamases [9,10,11,12,13,14,15,16,17,18,19,20,21,22,23]. In addition, *Serratia* species are also intrinsically resistant to nitrofurantoin, doxycycline, colistin and most of the aminoglycosides (with the exception of streptomycin and amikacin) [12,13,16,22,24]. Due to the clinical significance of their AmpC-β-lactamase-production, these pathogens are a part of the “SPICE” group (*Serratia*, *Pseudomonas*, indole-positive *Proteus*, *Citrobacter*, and *Enterobacter*) of bacteria [23,25]. To make matters worse, multidrug resistant strains (MDR), expressing plasmid-encoded (transmissible) extended-spectrum β-lactamases (ESBLs) or carbapenemases have emerged, where clinicians are left with very few and expensive (e.g., tigecycline, ceftazidime/avibactam) treatment options [26,27,28,29]. Therefore, it is no surprise that carbapenem-resistant *Enterobacter* species are a part of the “ESKAPE” pathogens, which are considered as the most concerning for the healthcare institutions worldwide [30,31,32].

The epidemiology and antibiotic susceptibility-patterns of urinary tract pathogens vary greatly by region. Therefore, the assessment of local data is essential to evaluate trends over time and to reflect on the national situation, compared to international data [33]. Additionally, knowledge of the relevant antibiotic susceptibility patterns of the major bacterial pathogens for UTIs is of utmost importance to allow for the optimal choice for antibiotic therapy [34,35,36]. The aim of this study was to assess and compare the resistance trends and epidemiology of different species of CES in UTIs (RECESUTI) in inpatients and outpatients at the Albert Szent-Györgyi Clinical Center (Szeged, Hungary) retrospectively, during a 10-year study period.

## 2. Materials and Methods

### 2.1. Study Design, Data Collection

This retrospective study was carried out using microbiological data collected from the period between January 1, 2008, and December 31, 2017, at the Institute of Clinical Microbiology (University of Szeged), which is the affiliated diagnostic microbiology laboratory of the Albert Szent-Györgyi Clinical Center, a primary-and tertiary-care teaching hospital in the Southern Great Plain of Hungary. The Clinical Center has a bed capacity of 1820-beds (1465 acute and 355 chronic beds, respectively) and annually serves more than 400,000 patients in the region, according to the data of the Hungarian National Health Insurance Fund (NEAK) [37]. Based on the data of the Hungarian Central Statistical Office (KSH), the ratio of the 0—14-year-old population in the region is around 15.3%, while people over 60 years of age represent around 21.2% of the regional population. Electronic search in the records of the MedBakter laboratory information system (LIS) for urine samples positive for *Citrobacter*, *Enterobacter*, and *Serratia* (CES) species was conducted.

Samples with clinically significant colony counts for CES (>10^5^ CFU/mL; however, this was subject to interpretation based on the information provided on the request forms for microbiological analysis and relevant international guidelines, e.g., presence of underlying conditions in the genitourinary tract) were included in the data analysis. Only the first isolate per patient was included in the study. However, isolates with different antibiotic-susceptibility patterns were considered as different individual isolates. In addition, patient data was also collected, which were limited to demographic characteristics (age and sex). The study was deemed exempt from ethics review by the Institutional Review Board, and informed consent was not required as data anonymity was maintained.

### 2.2. Identification of Isolates

Ten microliters of each uncentrifuged urine sample was cultured on UriSelect chromogenic agar plates (Bio-Rad, Berkeley, CA, USA) with a calibrated loop, according to the manufacturer’s instructions, and incubated at 37 °C for 24–48 h, aerobically. If the relevant pathogens presented in significant colony count, the plates were passed on for further processing. Between 2008–2012, presumptive phenotypic (biochemical reaction-based) methods and VITEK 2 ID (bioMérieux, Marcy-l’Étoile, France) were used for bacterial identification, while after 2013, this was complemented by matrix-assisted laser desorption/ionization time-of-flight mass spectrometry (MALDI-TOF MS; Bruker Daltonik Gmbh. Gr.). The methodology of sample preparation for MALDI-TOF MS measurements was described elsewhere [38,39]. Mass spectrometry was performed by the Microflex MALDI Biotyper (Bruker Daltonics, Germany) in positive linear mode across the m/z range of 2 to 20 kDa; for each spectrum, 240 laser shots at 60 Hz in groups of 40 shots per sampling area were collected. The MALDI Biotyper RTC 3.1 software (Bruker Daltonics, Germany) and the MALDI Biotyper Library 3.1 were used for spectrum analysis.

### 2.3. Antimicrobial Susceptibility Testing

Antimicrobial susceptibility testing (AST) was performed using the Kirby-Bauer disk diffusion method and E-test (Liofilchem, Abruzzo, Italy) on Mueller-Hinton agar (MHA) plates. In addition, for the verification of discrepant results, VITEK 2 AST (bioMérieux, Marcy-l’Étoile, France) was also utilized. The interpretation of the results was based on EUCAST breakpoints (http://www.eucast.org). *Staphylococcus aureus* ATCC 29213, *Enterococcus faecalis* ATCC 29212, *Proteus mirabilis* ATCC 35659, *Escherichia coli* ATCC 25922, and *Pseudomonas aeruginosa* ATCC 27853 were used as quality control strains.

To evaluate the resistance trends of isolated strains, ciprofloxacin (CIP), ceftriaxone (CRO), meropenem (MER), gentamicin (GEN; relevant in case of *Citrobacter* and *Enterobacter* spp.), and sulfamethoxazole/trimetoprim (SXT) were chosen as indicator antibiotics based on local antibiotic utilization data [22,40,41]. In addition, susceptibility data for doxycycline (DOX; relevant in case of *Citrobacter* and *Enterobacter* spp.) was available for the first half (2008–2012) of the study period, and for fosfomycin (FOS), data was available for the second half (2013–2017) of the study period. FOS susceptibility testing was not routinely performed. Instead, it was performed only in cases of extensive drug resistance or per request of the clinicians. During data analysis, intermediately susceptible results were grouped with and reported as resistant. Detection of extended-spectrum beta-lactamase (ESBL)-producing isolates was carried out based on EUCAST recommendations (http://www.eucast.org/resistance_mechanisms/).

### 2.4. Statistical Analysis

Descriptive statistical analysis (including means or medians with ranges and percentages to characterize data) was performed using Microsoft Excel 2013 (Redmond, WA, USA, Microsoft Corp.). Statistical analyses were performed with SPSS software version 24 (IBM SPSS Statistics for Windows 24.0, Armonk, NY, USA, IBM Corp.), using the χ^2^-test, Student’s *t*-test and Mann-Whitney U test. The normality of variables was tested using Shapiro-Wilk tests. *p* values < 0.05 were considered statistically significant.

## 3. Results

### 3.1. Demographic Characteristics, Sample Types

**Figure 1:**
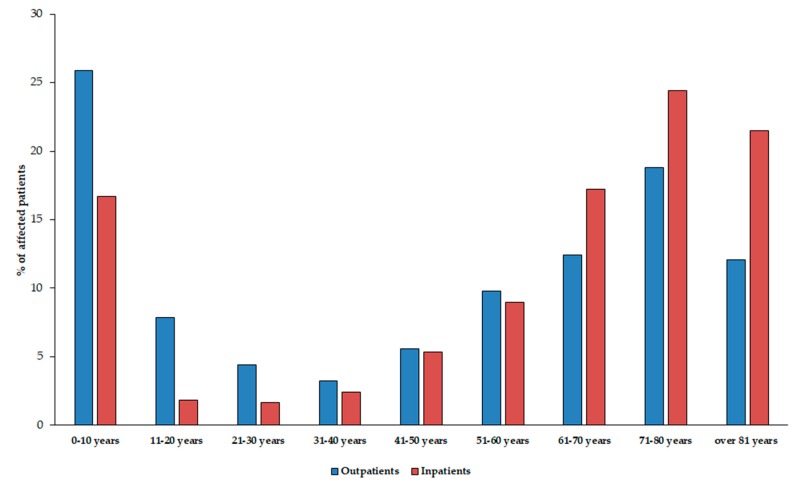
Age distribution of the affected patients in the outpatient and inpatient group.

The median age of affected patients was 56 years (range: 0.3–97) in the outpatient group with a female-to-male ratio of 1.1 (52.4% female), while in the inpatient group, these values were 68 years (range: 0.9–98) and 1.1 (52.4% female), respectively. The detailed age distribution of patients in both affected patient groups is presented in Figure 1. The difference in the age distribution of the two patient groups was statistically significant (*p* < 0.0001). Among the affected patients, the age groups under 10 years of age (outpatients: 25.7%, inpatients: 16.7%) and over 60 years of age (outpatients: 43.4%, inpatients: 63.1%) were the most numerous.

All (100%) samples received from outpatient clinics were voided (midstream) urine, while the sample distribution from the inpatient departments was the following: Catheter-specimen urine (47.1%), midstream urine (41.0%), first-stream urine (11.6%), and samples obtained through suprapubic bladder aspiration (0.3%).

### 3.2. Distribution of Citrobacter-Enterobacter-Serratia Isolates

**Figure 2:**
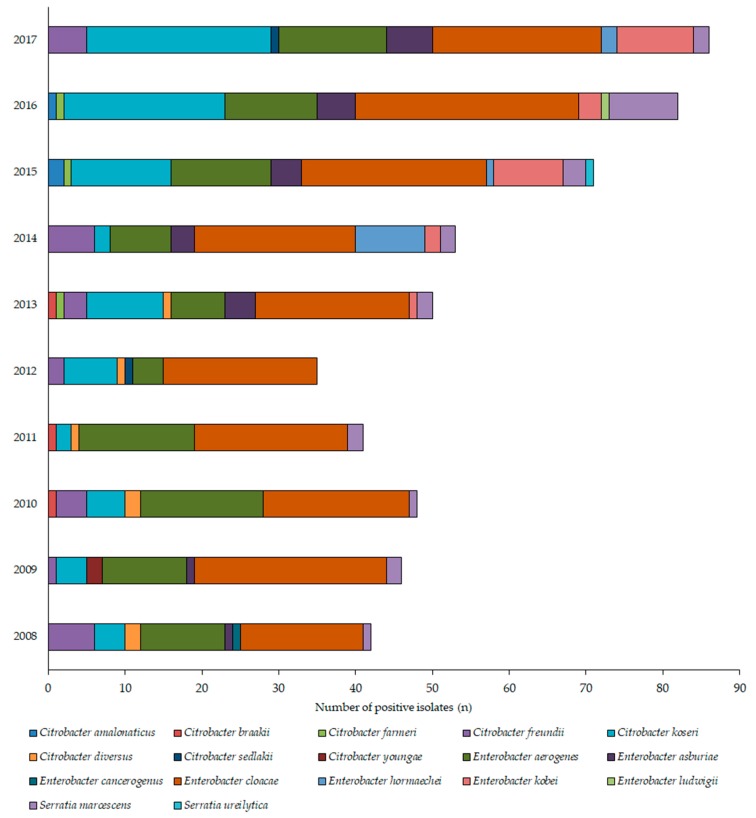
Frequency and species distribution of *Citrobacter*, *Enterobacter*, and *Serratia* (CES) isolates in outpatient samples (2008—2017).

**Figure 3:**
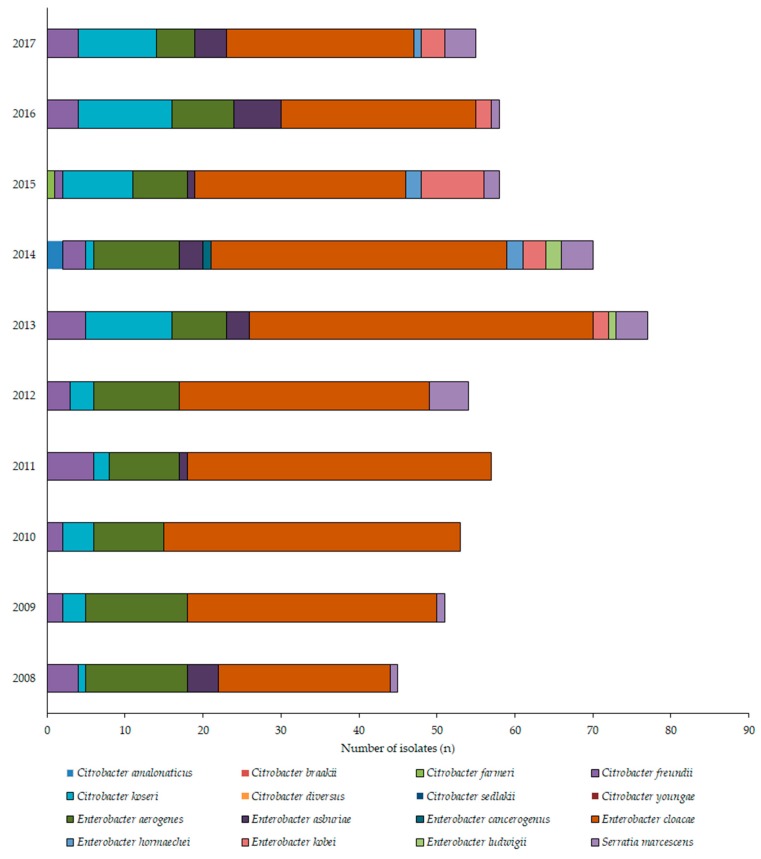
Frequency and species distribution of CES isolates in inpatient samples (2008—2017).

During the 10-year study period (1 January 2008–31 December 2017), the Institute of Clinical Microbiology received 21,150 urine samples from outpatient clinics and 19,325 samples from inpatient departments that turned out to be positive for a significant urinary pathogen. 578 *Citrobacter*/*Enterobacter*/*Serratia* isolates were obtained from inpatients and 554 from outpatients. Henceforth, out of the positive urine samples, these pathogens represented 2.6 ± 0.4% (range: 1.9–3.2%, lowest in 2012, highest in 2017) for outpatients, while 3.0 ± 0.4% (range: 2.5–3.8%, lowest in 2015, highest in 2013) of all positive urine samples; (*p* > 0.05). In both groups, *E. cloacae* (outpatients: 38.9%; inpatients: 55.5%), *E. aerogenes* (outpatients: 20.0%, inpatients: 15.9%), *C. koseri* (outpatients: 16.6%, inpatients: 9.7%), and *C. freundii* (outpatients: 4.9%, inpatients: 5.9%) were the most prevalent, while *Serratia* species accounted for 4.5% and 3.8% of the isolates, respectively. The epidemiology and total species distribution of outpatient and inpatient samples is presented in Figure 2 (inpatients) and Figure 3 (outpatients). In the inpatient group, 11 different species of CES were isolated, while in the outpatient group, the species distribution was more diverse, with 17 different species detected.

### 3.3. Antibiotic Susceptibility Trends among CES Isolates

The resistance trends of the isolates *Citrobacter*/*Enterobacter*/*Serratia* species against ciprofloxacin (CIP), ceftriaxone (CRO), gentamicin (GEN; regarding *Enterobacter* and *Citrobacter* species), doxycycline (DOX; regarding *Enterobacter* and *Citrobacter* species), fosfomycin (FOS), and sulfamethoxazole-trimethoprim (SXT) during the 10-year surveillance period are presented in Table 1; Table 2. The ratio of resistant strains in the inpatient group were significantly higher to CIP, CRO, GEN, and DOX (based on data from 2008–2012) (*p* = 0.0189, *p* = 0.0167, *p* = 0.0232, and *p* = 0.0342, respectively), but not in case of SUM and FOS (*p* > 0.05). In addition, resistance levels to the indicator antibiotics were significantly higher (*p* < 0.05) in the first half (2008–2012) of the study period in case of every drug (apart from DOX and FOS, where resistance data was not available throughout the 10-year period) (Table 1 and Table 2).

Overall, the highest levels of resistance were recorded for CRO (outpatients: highest in 2010, lowest in 2017; inpatients: highest in 2009, lowest in 2015), while the lowest for GEN (outpatients: highest in 2009, lowest in 2014; inpatients: highest in 2014, lowest in 2017). During the course of the study period, increasing resistance levels could be observed until 2009–2011, while around 2012–2014, a dramatic (5–25-times) decrease in the ratio of resistant strains was noted, except in the case of CRO. DOX-resistance levels were around 30% in the inpatient group and above 30% in the outpatient group between 2008–2012 (Table 2). FOS susceptibility-testing was performed in around 10% of the isolates. Resistance levels were between 20–57.1% for outpatients and 17.1–77.9% for inpatients (2013–2017). The ratio of ESBL-producing isolates was ranging between 0–9.8% (lowest in 2008–2009, highest in 2011) from outpatient samples and 0–29.1% (lowest in 2008–2009, highest in 2010) from inpatient samples; ESBL-positivity was detected more frequently in inpatient samples (*p* = 0.0152; Table 2). No meropenem-resistant isolates were recovered during the 10-year study period.

## 4. Discussion

*Enterobacterales* (the novel taxonomic designation of the *Enterobacteriaceae* family) are the most common (70–80%) cause of urinary tract infections (UTIs) in both community and healthcare settings [1,2,42,43]. Empiric antibiotic therapy should be selected based on local susceptibility profiles or a cumulative hospital antibiogram. Nevertheless, the choice of antimicrobial drugs should be revised after the specific antibiogram for the relevant urinary pathogen has become available [44,45]. Guidelines of the Infectious Diseases Society of America (IDSA) also recommend that one of the main factors of choosing empiric antibiotic therapy for UTIs is local resistance data, in addition to considering the history of the patient, drug allergies/intolerance and local/institutional drug availability [1,3,46]. In general, nitrofurantoin, sulfamethoxazole-trimethoprim and fosfomycin should be used for uncomplicated UTIs empirically (if local resistance levels do not exceed 20%), while for complicated UTIs or pyelonephritis, third generation cephalosporins (e.g., ceftriaxone), fluoroquinolones, aminoglycosides, or carbapenems should be used [1,3,46,47,48].

In the context of our study, the members of the *Citrobacter*, *Enterobacter*, and *Serratia* genera were causative agents in UTIs in around 2.5–3% of cases in both outpatient and inpatient settings. Although on first approach this may seem as relatively low prevalence, their clinical relevance should not be disregarded in either settings. There is a shortage of data available on the prevalence and resistance trends of CES isolates, and the available published evidence is usually in the form of larger multicenter or international surveillance studies (e.g., SENTRY Antimicrobial Surveillance Program): In these reports, the overall prevalence of CES isolates ranges between 0.5–18% in urinary tract infections [2,16,49,50,51]. Some reports suggest that *Citrobacter* and *Enterobacter* species are the third most common pathogens in UTIs, while in others, they are less frequently isolated than *Proteae* [2,16,49,50,51,52,53]. Based on the results of this retrospective survey, the most prevalent isolates at our tertiary-care center were *E. aerogenes* and *E. cloacae*; interestingly, the species distribution in the outpatient isolates was ~1.5-times higher, than in the outpatient group, while in the literature, the opposite is generally observed [49,51].

Regarding the local resistance levels, the results of the 10-year survey showed that there has been a pronounced decrease in the resistance rates of several antibiotics (cf. ceftriaxone) in the period of 2012–2014. In fact, some antibiotics had their lowest resistance rates in the last year (2017) of the study period. There was no single underlying event found that may be responsible for this local advantageous change in resistance levels, although the developments in antibiotic stewardship and stricter adherence to diagnostic and therapeutic guidelines–both at the Albert Szent-Györgyi Clinical Center and in the country in general from the 2010s–may have had a notable role [22,40,41,42,54,55]. The most concerning development is the resistance rates to third generation cephalosporins (exemplified by ceftriaxone in this survey), where even the lowest levels of resistance were around 20% in the outpatient group and close to 30% in the inpatient group. Resistance to β-lactam antibiotics should be considered as a serious issue, because in some vulnerable patient groups–like pregnant women and children, where many other antibiotic drugs cannot be used–they are the first-choice agents [56]. In some cases (e.g., an ESBL-positive *Serratia*), where special patient groups are affected, carbapenem antibiotics remain the singular choice of drugs. In Hungary (and, specifically, in the southern region of the country), the *bla*_CTX-M_ group is the most prevalent, which is associated with carrying resistance determinants to quinolones and aminoglycosides in addition to the relevant β-lactam antibiotics [57]. It is worth noting that if the isolate is resistant to quinolones, sulfamethoxazole-trimethoprim and fosfomycin, there are basically no orally available therapeutic options left for the treatment of CES infections. In this case, treatment needs to be carried out in an inpatient setting, or through outpatient parenteral antibiotic therapy (OPAT), utilizing aminoglycosides (marginally effective against *Serratia*), third–fourth generation cephalosporins or carbapenems [35,58,59]. The differences in the resistance rates among inpatient and outpatient samples overall may have been influenced by the species distribution as well (Figure 2; Figure 3). In the inpatient group, *E. cloacae* was the most predominant isolate, the species distribution in the inpatient isolates was more balanced.

The emergence and spread of ESBL-and/or carbapenemase-producing *Enterobacterales* is a serious concern in any case, especially if the pathogens in question also have additional intrinsic resistance mechanisms. Consequently, the therapeutic armamentarium becomes very limited. Tigecycline has activity against ESBL-producing bacteria, however, due to its pharmacokinetics, it is not ideal for the treatment of urinary tract infections [60]. Colistin has recently become the “last resort” antibiotic in the therapy of Gram-negative MDR infections: it has shown clinical effectiveness where other options were not available, however, this drug cases severe nephrotoxicity and neurotoxicity. Therefore, it should be reserved for infections caused by carbapenem and aminoglycoside-resistant infections [61,62]. In addition, *Serratia* species are colistin resistant [22]. Novel antibiotics (e.g., ceftolozane-tazobactam, ceftazidime-avibactam, ceftaroline-avibactam, delafloxacin etc.) offer new hope in the effective therapy of all sorts of infections caused by MDR Gram-negative pathogens, although due to their exorbitant price and limited clinical experience, it is questionable how long will it take for these drugs to be acknowledged in the routine therapeutic protocols [28,63,64,65,66].

Some limitations of this study must be acknowledged. First, the study design is retrospective and due to the inability to access the medical records of the individual patients affected, the correlation between the existence of relevant risk factors and underlying illnesses (apart from age, inpatient/outpatient status, and catheterization) and *Citrobacter*/*Enterobacter*/*Serratia* UTIs could not be assessed. The age-associated incidence in isolation of CES may also reflect (at least in part) the high rate of bacteriuria in the elderly population. Furthermore, molecular characterization of the genetic background of resistance in the individual isolates was not performed, only to the extent of presence/absence of ESBLs. There is a risk of selection bias, as studies describing the prevalence of infectious diseases and resistance trends are tertiary-care centers, which generally correspond to patients with more severe conditions or underlying illnesses [67].

## 5. Conclusions

This study presents the epidemiological trends and resistance levels of *Citrobacter*/*Enterobacter*/*Serratia* associated with of urinary tract infections (UTIs) in Hungary over a long surveillance period (10 years), demonstrating the gradient of change in the resistance levels regarding various antibiotics. To the best of our knowledge, this is the first and longest-spanning study reporting on the prevalence and susceptibility patterns of CES pathogens (and UTIs caused by these uropathogens by proxy) in Hungary. Their higher prevalence in patients with advanced age (over 60 years of age) is in line with the findings in literature, while the type of setting (inpatient/outpatient) did not have an effect on their isolation frequency.

The emergence of this usually rare organism as an increasingly common urinary pathogen is alarming. During the current study period, the susceptibility of CES bacteria showed an advantageous trend (excluding resistance to β-lactam antibiotics; nevertheless, this trend is only sustainable through strict adherence to infection control practices and relevant therapeutic and diagnostic guidelines). As the therapeutic options are largely limited in the current antibiotic resistance climate, energies should be put into the prudent use of antibiotics. In addition, due to the potential of these pathogens to cause nosocomial outbreaks (usually in vulnerable patient groups), stern and continuous surveillance is required on both institutional and on a national level.

## Figures and Tables

**Table 1:** Percentage of resistant strains to indicator antibiotics from inpatient and outpatient departments (2008–2017).

		2008	2009	2010	2011	2012	2013	2014	2015	2016	2017	Overall (±SE)	Statistics
**CIP R%**	*Outpatient*	*2.4*	23.9	18.8	**26.9**	8.6	7.0	5.6	8.7	6.1	5.8	11.4 ± 2.7	*p* = 0.0189
	*Inpatient*	36.4	37.3	45.3	**54.6**	44.3	40.8	8.6	8.5	6.8	*3.9*	28.6 ± 6.1	
**CRO R%**	*Outpatient*	30.6	47.8	**47.9**	29.3	25.7	24.0	24.1	24.4	24.4	*19.5*	29.8 ± 3.2	*p* = 0.0167
	*Inpatient*	65.9	**72.5**	64.2	61.8	56.8	43.4	28.6	*27.1*	27.1	27.	47.5 ± 5.9	
**GEN R%**	*Outpatient*	4.9	**19.6**	16.7	17.9	5.7	8.3	*1.9*	3.2	2.6	2.4	8.3 ± 2.2	*p* = 0.0232
	*Inpatient*	**51.9**	48.0	45.3	47.3	27.5	11.1	7.6	5.3	5.3	*5.*	25.4 ± 6.5	
**SUM R%**	*Outpatient*	16.7	21.4	14.6	**24.4**	15.7	12.0	3.7	8.7	3.7	*1.1*	12.2 ± 2.5	n.s. (*p* = 0.0778)
	*Inpatient*	**50.0**	49.0	37.3	31.8	16.1	13.2	14.3	10.2	10.2	*1.9*	23.5 ± 5.5	

Values in italics represent the lowest resistance levels, boldface (peak) values correspond to the highest resistance levels in the study period; n.s.: Not significant.

**Table 2:** Percentage of resistant strains to indicator antibiotics from inpatient and outpatient departments.

		2008	2009	2010	2011	2012	2013	2014	2015	2016	2017	Overall (±SE)	Statistics
**DOX R%**	*Outpatient*	31.7	30.0	*27.7*	**38.5**	35.7						32.7 ± 1.9	*p* = 0.0342
	*Inpatient*	**48.9**	44.5	*33.0*	39.3	40.0						41.1 ± 2.7	
**FOS R%**	*Outpatient*						10.0%	7.4%	8.7%	12.2%	8.1%	9.3 ± 0.8	n.s. (*p* = 0.454)
	*Inpatient*						7.7%	15.7%	20.3%	15.3%	9.8%	13.8 ± 2.1	
**ESBL %**	*Outpatient*	0.0	0.0	8.8	9.8	2.9	8.0	1.9	4.8	1.2	2.3	3.7 ± 1.2	(*p* = 0.0152)
	*Inpatient*	0.0	0.0	28.3	29.1	7.8	7.9	11.4	1.7	1.7	5.9	10.9 ± 3.4	

Values in italics represent the lowest resistance levels, boldface (peak) values correspond to the highest resistance levels in the study period; n.s.: Not significant.
